# Graphene Oxide Research: Current Developments and Future Directions

**DOI:** 10.3390/nano15070507

**Published:** 2025-03-28

**Authors:** Meiqiu Zhan, Minjie Xu, Weijun Lin, Haijie He, Chuang He

**Affiliations:** 1 School of Civil Engineering, Jilin Jianzhu University, Changchun 130119, China; meiqiuzhan@foxmail.com (M.Z.); xuminjie0510@gmail.com (M.X.); 2 School of Civil Engineering and Architecture, Taizhou University, Taizhou 317000, China; 3 Taizhou Construction Engineering Group Co., Ltd., Taizhou 317000, China; 13620707291@163.com; 4 College of Civil and Architectural Engineering, Zhejiang University, Hangzhou 310027, China

**Keywords:** graphene oxide, synthesis, applications, scientometrics, CiteSpace

## Abstract

Graphene oxide (GO), a pivotal derivative of graphene, has revolutionized nanotechnology with its tunable physicochemical properties and interdisciplinary applications in energy storage, environmental remediation, and biomedicine. Despite its exponential research growth, existing reviews remain fragmented, lacking holistic insights into evolving synthesis–application linkages, global collaboration patterns, and emerging convergence trends. This study bridges these gaps through a scientometric analysis of 14,124 peer-reviewed articles (2022–2025) from the Web of Science Core Collection, utilizing CiteSpace for co-occurrence network mapping, burst detection, and cluster analysis. Key findings reveal (1) a thematic shift from traditional synthesis optimization (e.g., Hummers’ method) toward driven material design and sustainable applications like GO membranes for water purification; (2) China’s dominance in publication output (38.5%) contrasts with the U.S. and Europe’s leadership in global collaborations; (3) interdisciplinary journals such as *Chemical Engineering Journal* (centrality: 0.25) and emerging clusters like “circular economy” signal transformative priorities; and (4) critical gaps in scalability, ecological safety, and cost-effective production hinder industrial translation. This work provides a roadmap for aligning research with sustainability goals, fostering global partnerships, and accelerating innovations in scalable nanotechnology.

## 1. Introduction

Graphene is celebrated for its exceptional mechanical strength and electrical conductivity. Its high production cost and processing challenges have driven extensive research into graphene oxide as a cost-effective alternative. Graphene oxide (GO), an oxidized derivative of graphene, has emerged as a pivotal material in nanoscience and nanotechnology due to its unique physicochemical properties and versatile applications [1,2,3,4,5]. Structurally, GO consists of a two-dimensional honeycomb lattice of carbon atoms functionalized with oxygen-containing groups [6], such as hydroxyl, epoxy, and carboxyl moieties, which confer hydrophilicity and chemical tunability—distinguishing it from pristine graphene. These oxygen functionalities enable three critical attributes: (i) aqueous processability, allowing stable colloidal dispersions for solution-based processing; (ii) pH-dependent surface charge (−30 to +25 mV zeta potential), critical for electrostatic interactions in pollutant adsorption; and (iii) reactive sites for covalent/noncovalent functionalization to tailor properties for specific applications [7,8]. The nomenclature distinction between GO and reduced graphene oxide (rGO) hinges on the degree of oxygen removal: when GO undergoes chemical, thermal, or photochemical reduction to restore sp^2^ hybridized networks (C/O ratio > 8:1), the resulting material transitions from insulating GO (~10^−3^ S/m) to conductive rGO (>10^2^ S/m), while retaining residual oxygen groups (~5–10 wt%) for interfacial compatibility [9]. GO synthesis predominantly follows graphite oxidation protocols, including Brodie’s, Staudenmaier’s, Hofmann’s, and Hummers’s protocols, with Hummers’ method and its modified variants being the most widely adopted due to scalability and efficiency [10,11,12,13,14]. These methods yield GO with adjustable carbon-to-oxygen ratios, enabling tailored properties for specific applications. For energy storage devices (e.g., supercapacitors), GO’s high theoretical surface area (~2600 m^2^/g) facilitates ion adsorption at electrode interfaces [15], while its oxygen groups enable heteroatom doping during rGO synthesis to enhance pseudocapacitance [5,10]. In environmental remediation, edge-localized carboxyl groups (-COOH) provide selective binding sites for heavy metal cations (e.g., Pb^2+^ and Hg^2+^) via chelation, achieving > 90% removal efficiency. Biomedical applications exploit GO’s biocompatibility and π-π stacking interactions [16,17,18]: its planar structure allows ~90% drug loading capacity (e.g., doxorubicin), while oxygen groups enable PEGylation for prolonged blood circulation [19].

The rapid advancement of GO research has spurred a proliferation of reviews addressing its multifaceted synthesis and application landscapes, though the existing literature predominantly adopts segmented approaches reflecting its interdisciplinary nature. From a methodological perspective, studies have dissected conventional synthesis techniques, emphasizing optimization strategies to enhance oxidation efficiency and scalability, as seen in critical analyses of Hummers’s method and its modified variants for improving structural uniformity and reproducibility [13,20,21]. A mesoscale focus on hybrid systems explores GO’s integration with materials such as metal–organic frameworks (MOFs) or noble metal nanoparticles, highlighting synergistic functionalities, particularly in Au/Ag-rGO composites tailored for catalytic, sensing, and biomedical applications through in situ or ex situ hybridization strategies [22,23]. Concurrently, application-oriented reviews systematically categorize GO’s roles in environmental remediation, such as pollutant adsorption; and biomedical innovations, like drug delivery platforms and antimicrobial coatings [24], while niche derivatives, such as graphene oxide nanoscrolls (GONSs), highlight fabrication challenges, structural advantages, and emerging applications in energy storage and gas sensing [24]. Despite these advancements, current syntheses exhibit critical limitations, including fragmented emphases on isolated domains that obscure holistic trends and knowledge evolution, along with gaps in unifying scalable production methods with application-driven requirements. While industrial-scale GO synthesis has been conceptually outlined [25,26], systematic evaluations linking production innovations to real-world performance in sectors like energy or biomedicine remain underdeveloped. Furthermore, emerging paradigms such as machine learning-assisted material design and circular economy-aligned GO recycling demand deeper integration into mainstream discourse.

As an interdisciplinary branch of informatics, scientometrics offers a robust framework for quantitatively analyzing publication patterns to decode emerging trends and knowledge architectures within research domains [27,28,29]. By leveraging advanced visualization tools, this approach transforms voluminous scientific literature into interactive maps, enabling systematic exploration of collaborative networks, co-citation dynamics, and keyword evolution. Conventional scientometric and bibliometric [30,31] software programs, such as HistCite and RefViz, are limited to word frequency analysis or keyword grouping, and VOSviewer excels in mapping prolific contributors but lacks relational depth. CiteSpace stands out as a versatile Java-based application uniquely equipped to address these gaps [32,33]. Its capabilities in burst detection, betweenness centrality analysis, and heterogeneous network visualization allow researchers to pinpoint pivotal turning points, interdisciplinary convergences, and frontier topics within complex datasets [34,35,36]. GO research over the past three years not only reveals the structural evolution of fields but also bridges disciplinary silos, offering a holistic view of knowledge growth. In the context of graphene oxide research, where exponential publication growth necessitates tools to synthesize fragmented advancements, CiteSpace proves indispensable [32,33]. It empowers the identification of core themes, such as scalable synthesis innovations, hybrid material synergies, and emerging applications in biomedical or environmental sectors, while forecasting transformative trends at the intersection of computational design and sustainable material cycles. However, to date, there has been no study utilizing CiteSpace for a comprehensive bibliometric analysis of graphene oxide research, particularly in mapping interdisciplinary synthesis–property–application linkages and emerging convergence trends.

Building upon the scientometric foundation established in prior sections, this review systematically investigates the intellectual structure, evolutionary trajectories, and interdisciplinary frontiers of GO research over the past three years. A comprehensive dataset comprising 15,066 peer-reviewed articles and conference proceedings (2022–2025) was extracted from the Web of Science Core Collection (WOSCC) database, ensuring broad coverage of synthesis, functionalization, and application studies across disciplines. Utilizing CiteSpace’s advanced analytical modules, this study first maps the global collaborative landscape by identifying leading countries, institutions, and authors, highlighting knowledge dissemination patterns and interdisciplinary synergies. Key journals and highly cited references are analyzed to delineate authoritative knowledge sources and foundational theories. Subsequent keyword co-occurrence and clustering analyses reveal emergent research hotspots, such as scalable synthesis methods, GO–polymer hybrids, and biomedical applications, while timeline visualization traces thematic shifts from early oxidation optimization to contemporary artificial intelligence-driven material design. Burst detection further identifies frontier topics, including circular economy-aligned recycling strategies and machine learning-guided property prediction. Citation network analysis uncovers transformative works bridging fundamental chemistry and applied nanotechnology, emphasizing cross-domain knowledge transfer. This review bridges these gaps through a scientometric lens, decoding interdisciplinary trajectories, identifying convergences, and forecasting frontier domains to crystallize the current knowledge architecture and empower strategic navigation of this dynamic field.

## 2. Data and Methods

### 2.1. Data Sources and Selection Process

The Web of Science Core Collection (WoSCC), a multidisciplinary citation database encompassing high-quality journals across scientific disciplines, was selected to retrieve literature on GO synthesis and applications [32,33]. A targeted search strategy was executed on 5 February 2025, covering publications from 2022 to 2025, using the following Boolean query: TI = (“graphene oxide*” OR GO OR “graphite oxide” OR “oxidized graphene”) AND TS = (synthes* OR preparation OR fabricat* OR manufacturing OR development OR produc* OR application* OR use* OR utili* OR function* OR technology). This approach combined title-restricted keywords for specificity (e.g., “graphene oxide” variants) with topic-expanded terms in abstracts/keywords to capture synthesis techniques and application contexts. Lexical variations were addressed using wildcards (*), while article types were limited to peer-reviewed articles and reviews in English.

The initial search yielded 15,066 valid records (13,219 articles; 362 reviews), refined by removing duplicates, non-English entries, and non-research document types (e.g., books and patents). Full metadata, including titles, abstracts, keywords, and citation references, were exported in plain-text format for bibliometric analysis through CiteSpace [32,33]. The final dataset focuses exclusively on publications addressing GO synthesis methodologies, functionalization innovations, and practical applications across material science, biomedicine, and energy technologies.

### 2.2. Analysis Method

This study employed CiteSpace 6.1.R4 to conduct bibliometric analyses, focusing on the evolution of GO research from 2022 to 2025. Following data import, raw records were filtered to retain research articles and reviews, yielding 14,124 publications as the analytical corpus. Temporal slicing was configured with 1-year intervals (2022–2025), and node types were set to keywords to map conceptual trends. The pruning parameters included the Pathfinder algorithm to simplify network connectivity while retaining critical links, and a threshold setting for each time slice was defined as the top 10% of high-frequency keywords, capped at 100 keywords per slice to balance granularity and interpretability [32].

Co-occurrence networks were constructed using aggregated keyword frequencies to identify interdisciplinary clusters and emerging themes, while centrality metrics highlighted pivotal nodes bridging distinct research domains [33,36]. Burst detection algorithms were applied to pinpoint abrupt increases in keyword usage, signaling paradigm shifts or technological breakthroughs. Time-zone visualization was generated to illustrate temporal interdependencies among research fronts, and cluster labeling ensured semantic coherence by merging synonymous terms. Default parameters governed smaller network components (e.g., node size scaling and link strength), ensuring methodological consistency with prior bibliometric studies.

## 3. Scientometric Analysis and Visualization

The analyzed corpus comprises 14,124 publications (all designated as research articles), spanning 2142 journals, with contributions from 58,633 authors, 34,932 institutions, and 706 countries or regions. This broad coverage underscores the global and interdisciplinary nature of graphene oxide research between 2022 and 2025, reflecting substantial collaboration across academia and industry. The extensive participation from diverse geographical and institutional entities highlights both the scientific significance of GO and its wide-ranging applicability in fields such as materials science, energy storage, and biomedicine.

### 3.1. Trend Analysis of Issuance

**Figure 1:**
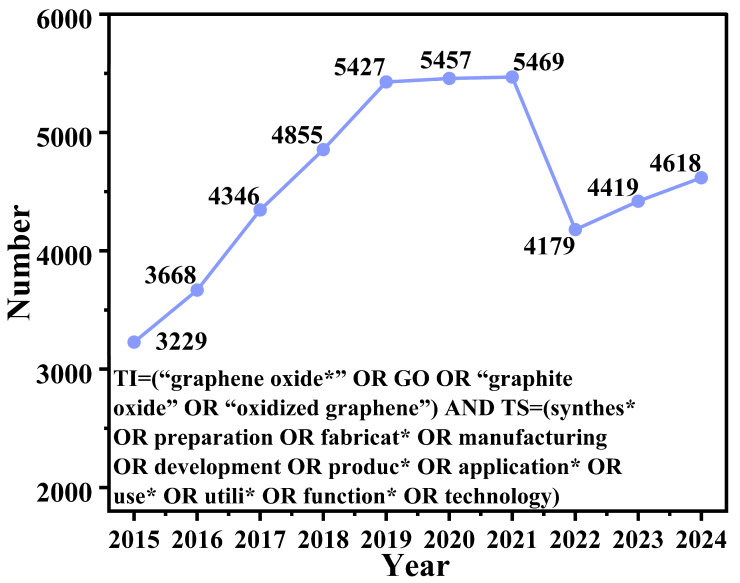
Annual publication trends from 2015 to 2024.

Figure 1 illustrates annual publication trends in graphene oxide research from 2015 to 2024 based on peer-reviewed articles containing “graphene oxide” and related preparation/application terminologies. Overall, this field exhibits dynamic yet sustained growth, characterized by steady expansion, followed by strategic recalibration. The data reveal two distinct phases:

The initial phase (2015–2021) demonstrates vigorous momentum with an 81.3% growth in annual publications, rising from 3229 to a peak of 5469 publications. This seven-year period witnessed consistent year-over-year increases averaging 4.1% annually, peaking in 2021. A strategic recalibration phase (2021–2024) follows, marked by an 18.8% publication decline from peak levels yet maintains outputs exceeding early-phase values. Despite the reduction to 4618 publications in 2024, annual production remains 43.0% higher than 2015 baselines. Notably, the 2024 output surpasses the pre-peak plateau (e.g., 2018–2019: 4855–5427), suggesting maturation rather than decline. Two critical observations emerge: first, the 2022–2023 stabilization (4179–4419 publications) aligns with typical 12–24-month development cycles for high-impact discoveries; second, incomplete 2024 data collection likely underrepresents actual annual outputs.

The trajectory collectively confirms graphene oxide’s enduring scientific relevance. While initial exponential growth transitions to strategic consolidation, publication volumes remain historically elevated (2024 volume ≈ 3.8 × the 2018 benchmark). This pattern reflects paradigm shifts toward quality-driven research, with increasing emphasis on industrial translation over exploratory synthesis. Future investigations should prioritize optimizing characterization protocols and scaling sustainable production methods to sustain leadership in this critical nanomaterial domain.

### 3.2. Analysis of Article Output Characteristics

Collaboration analysis provides a visualized framework to elucidate cooperative networks within GO research, revealing partnerships among authors, institutions, and countries. This approach identifies key contributors driving innovation (e.g., leading universities or laboratories) and maps the structural dynamics of research clusters, offering insights into thematic focus areas such as nanocomposite development, catalytic applications [37,38], or biomedical engineering. By analyzing co-authorship patterns, dominant collaborative hubs and emerging interdisciplinary frontiers are highlighted. Such analysis not only aids in pinpointing influential stakeholders but also fosters global scientific dialogue by encouraging underrepresented regions or institutions to engage actively. The results will equip researchers with actionable strategies to optimize partnerships and accelerate breakthroughs in GO-related technologies.

The visualization employs chromatic and geometric encodings to represent temporal and structural attributes of nodes within the research network. Each node transitions in hue from cool tones (e.g., blue, 2022) to warm tones (e.g., red, 2025), mapping the temporal evolution of its scholarly activity. Nodes encircled by purple rings indicate entities with elevated betweenness centrality (normalized scores 0–1) [32,33], where the ring thickness scales proportionally to their intermediary role in bridging disconnected clusters. A centrality score exceeding 0.1 (denoted by thicker rings) typically correlates with critical junctures or knowledge hubs in the domain. Concurrently, edge weights between nodes reflect the frequency of collaborative interactions, while node size and perimetric color bandwidth encode publication metrics: larger diameters signify higher cumulative output, whereas thicker concentric bands represent enhanced annual productivity. This dual-layered encoding facilitates rapid identification of both prolific contributors and temporally dynamic actors within the network topology [32,33].

#### 3.2.1. Country or Regional Analysis

**Figure 2:**
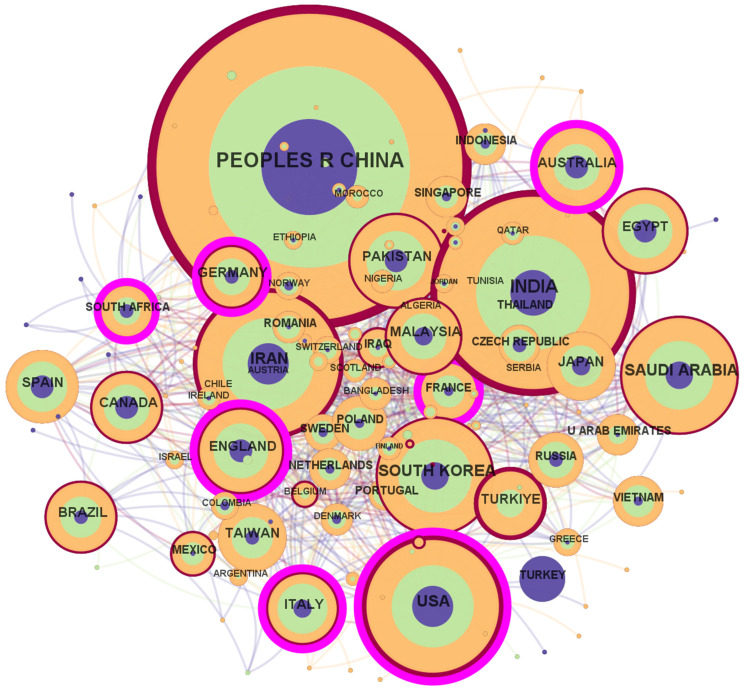
Distribution and cooperation of papers from diverse countries.

Through a statistical analysis of contributions across countries and institutions, we identified pivotal stakeholders in global GO research and their collaborative dynamics. From 2022 to 2025, 103 countries and 289 institutions participated in GO-related studies. Figure 2 displays the geographic distribution and cooperation patterns, while Table 1 ranks the top contributors by publication volume. China dominates the field with 5437 articles, accounting for 38.5% of total publications—far surpassing second-ranked India (2227 articles) and the United States (1053 articles). Chinese research primarily focuses on developing high-performance functional composites for advanced applications. Key innovations include multifunctional MIL-88A/rGO aerogels (Nanjing University) with −58.1 dB microwave absorption for stealth technology [39], and ultrafine metal oxide/rGO nanocomposites (East China University) achieving 225 L m^−2^ h^−1^ bar^−1^ filtration flux [20]. Despite this technical leadership, China’s low centrality (0.00) highlights underutilized potential for international collaboration. India’s output (2227 articles) emphasizes cost-effective synthesis and biomedical-energy applications of rGO–metal oxide composites. Notable advancements span Presidency University’s ppb-level rGO-ZnO/Fe_3_O_4_ gas sensors, Sikkim Manipal Institute’s MO/rGO supercapacitors (720 F/g specific capacitance), and Birla Institute’s PEG-functionalized GO nanoparticles for targeted drug delivery (80% loading efficiency) [40,41,42]. India’s strength in low-cost, sustainable synthesis positions it as an ideal partner for scalability-focused collaborations. Iran, with a substantial output of 1161 articles, demonstrates significant research activity concentrated in energy conversion and optoelectronic systems. Key contributions include Tarbiat Modares University’s Fe_2_Ni MIL-88B/rGO electrocatalysts (264 mV OER overpotential, outperforming IrO_2_) and Azad University’s RGO-ZnO nanocomposites, achieving a nonlinear refractive index of 31.9 × 10^−10^ cm^2^/W [43,44,45], critical for photonic device development. Iran’s growing publication volume underscores its emerging role in specialized domains, like electrocatalysis and renewable energy integration. The analysis reveals a pressing need to align publication volume with collaboration quality.

Table 2 highlights countries with high network influence, such as EU member states Italy (centrality: 0.17) and Germany (centrality: 0.17), and Australia (centrality: 0.10), which actively bridge collaborative clusters. Notably, the U.S. maintains strong ties with England, Australia, and Germany, while Saudi Arabia (centrality: 0.09) and South Korea (centrality: 0.06) serve as critical connectors in Middle Eastern and Asian networks, respectively. These results reveal a dichotomy: while China leads in quantity, Western nations and their allies drive interdisciplinary collaboration, particularly in areas like enhanced GO synthesis and sustainable material applications [46,47,48,49]. The data underscore the need for broader global partnerships to accelerate innovation in this strategically vital field.

#### 3.2.2. Institution Analysis

Research on GO encompassed contributions from 289 institutions globally between 2022 and 2025. Table 3 highlights the top 10 productive institutions, dominated by the Chinese Academy of Sciences, with 463 publications, accounting for approximately 9.6% of total contributions, followed by the Egyptian Knowledge Bank (EKB) (414 publications) and the Indian Institute of Technology System (284 publications). Notably, national research networks in China, India, and Saudi Arabia drive high output, with King Saud University (258 publications) exhibiting exceptional centrality (0.22), indicating its pivotal role in bridging interdisciplinary collaborations (Table 4).

**Figure 3:**
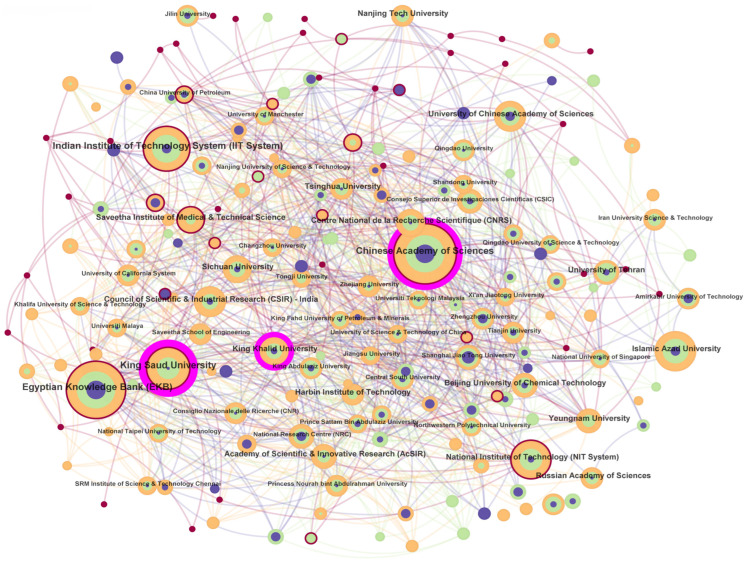
Distribution and cooperation of papers published by research institutions.

Figure 3 illustrates institutional collaboration patterns, analyzed via CiteSpace, where node size correlates with publication volume, and line thickness reflects partnership strength. Despite China’s dominance in quantity, institutions like King Saud University (centrality: 0.22) and Italy’s Centre National de la Recherche Scientifique (CNRS) (centrality: 0.08) serve as critical connectors, fostering cross-regional linkages. For instance, the Chinese Academy of Sciences collaborates extensively with Soochow University and Shanghai Jiao Tong University, while also engaging with international hubs in Korea and the USA [50].

The University of Chinese Academy of Sciences (143 publications, centrality: 0.02) and King Khalid University (127 publications, centrality: 0.13) further underscore the synergy between academic output and network influence. Institutions like Islamic Azad University (233 publications, centrality: 0.00) prioritize localized research with limited global ties, contrasting sharply with high-centrality Western counterparts.

Collaborative efforts were denser at the institutional level than nationally, particularly in developing GO-based technologies, like fire-resistant coatings and photocatalytic nanocomposites. For example, collaborative studies between the Chinese Academy of Sciences and University of Southern Queensland advanced flame-retardant GO composites, while partnerships involving Alexandria University and King Saud University optimized GO-based water purification systems [51]. These collaborations highlight the critical role of institutional hubs in translating GO research into scalable solutions for energy storage, environmental remediation, and biomedical engineering [10].

#### 3.2.3. Journal Co-Citation Analysis

The co-citation analysis of scholarly journals in GO research identifies key platforms driving innovation and knowledge exchange. As summarized in Table 5 and Table 6, *ACS Applied Materials & Interfaces* [52] (6807 citations) and *RSC Advances* (6707 citations) lead in citation frequency, alongside *Carbon* (6402 citations) and *Chemical Engineering Journal* (6071 citations), which dominate due to their focus on nanomaterials synthesis, device integration, and environmental applications. Notably, *RSC Advances* exhibits the highest centrality (0.36), highlighting its role as a bridge for interdisciplinary research despite its moderate impact factor (4.036). In contrast, high-impact journals like *ACS Nano* (5452 citations) and *Advanced Materials* (4528 citations) maintain prominence in foundational studies, such as GO-based energy storage and optoelectronics [53].

Highly co-cited journals often combine broad scope and high impact factors (>7). For instance, *Chemical Engineering Journal* (centrality: 0.25) serves as a hub for breakthroughs in GO-based water purification and catalytic degradation. Open-access journals like *RSC Advances* and *Scientific Reports* (5071 citations) achieve significant visibility through accessibility and rapid publication, while subscription-based journals like *Advanced Materials* attract seminal studies through academic prestige. Specialized platforms, such as *Journal of Colloid and Interface Science* (4231 citations) and *Electrochimica Acta* (centrality: 0.26), advance niche innovations—exploring GO’s surface chemistry and electrochemical applications in supercapacitors and sensors [54].

**Figure 4:**
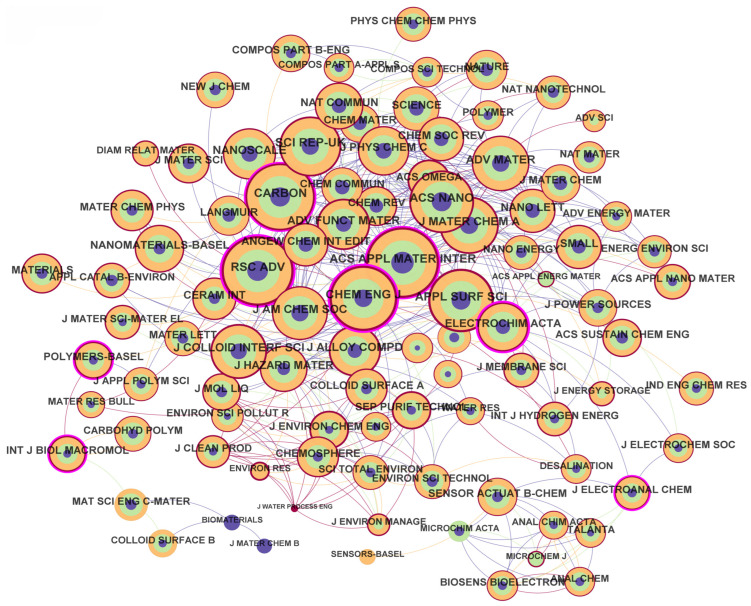
Distribution and cooperation of cited journals.

The co-citation network (Figure 4), comprising 2531 nodes and 9736 links, reveals GO research’s deep integration with diverse fields, from environmental science to flexible electronics. This interdisciplinary synergy is not merely thematic but methodological: Innovations in GO-based nanocomposites now require collaboration across materials science, chemical engineering, and computational modeling. Future advancements will depend on transcending traditional disciplinary boundaries, engaging journals in energy systems [55], biomedicine, and even social sciences to unlock GO’s full potential. This cross-pollination of ideas, epitomized by hybrid applications and novel methodologies, will remain central to sustaining the field’s momentum.

#### 3.2.4. Subject Analysis

**Figure 5:**
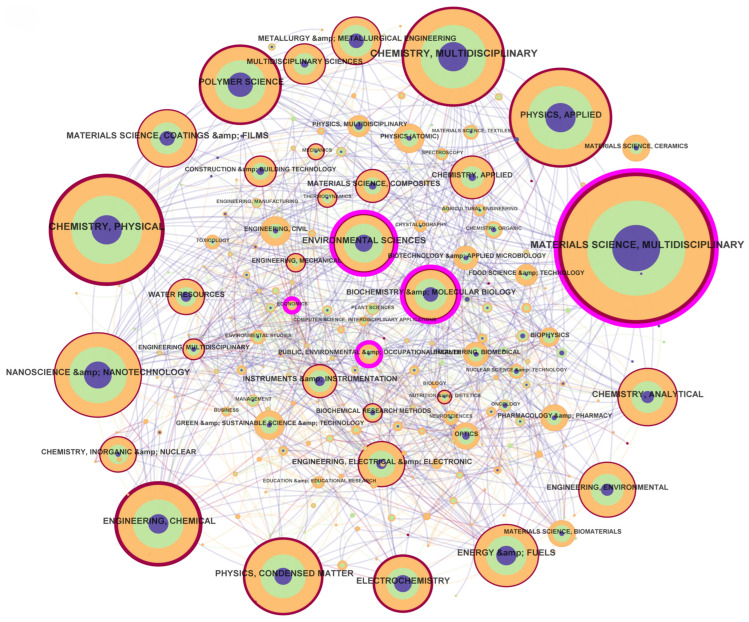
Disciplinary network.

Through the co-occurrence analysis of disciplinary categories (Figure 5), a robust interdisciplinary network emerges, reflecting the collaborative nature of electrochemiluminescence research. Table 7 highlights the top 10 contributing disciplines, underscoring their centrality and thematic dominance. Materials science—multidisciplinary leads with 3954 publications (centrality: 0.12), serving as the backbone of innovation through the design and synthesis of advanced electrochemiluminescent materials, such as quantum dots and nanocomposites. Chemistry—physical (2425 publications) and chemistry—multidisciplinary (1875 publications) further anchor the field, focusing on reaction mechanisms, luminescence efficiency optimization, and analytical method development.

The network reveals critical interdisciplinary bridges: nanoscience and nanotechnology (centrality: 0.07), and energy and fuels (centrality: 0.08) act as pivotal connectors between materials science and applied technologies. For instance, nanotechnology drives breakthroughs in electrode modification and signal amplification [26], while energy-focused studies explore electrochemiluminescence in battery health monitoring and fuel cell diagnostics. Physics—applied (1806 publications) and engineering—chemical (1447 publications) contribute to device integration and scalable fabrication processes, enhancing the transition from lab-scale research to industrial applications.

Burst analysis of emerging disciplines (Table 7) indicates dynamic shifts in research focus. While materials science—multidisciplinary, and energy and fuels exhibit sustained influence, short-lived bursts in chemistry—multidisciplinary (1 year) suggest rapid but transient interest in cross-methodological innovations, such as hybrid techniques combining electrochemiluminescence with spectroscopy or bioimaging. Conversely, disciplines like polymer science (1221 publications) show steady growth, reflecting their role in developing flexible, biocompatible luminescent polymers for wearable sensors and medical diagnostics [56,57].

The disciplinary network (Figure 5) emphasizes the necessity of cross-domain collaboration. For example, chemistry—analytical (715 publications) synergizes with medical laboratory technology to advance point-of-care diagnostics, leveraging electrochemiluminescence’s high sensitivity for pathogen detection. similarly, physics—condensed matter (1154 publications) bridges fundamental studies on charge-transfer kinetics with materials engineering, enabling tailored nanostructures for enhanced luminescent output. Future advancements will depend on deepening ties between niche disciplines (e.g., inorganic and nuclear chemistry) and applied fields, fostering innovations that address challenges in environmental monitoring, energy storage, and personalized medicine [58].

### 3.3. The Key Areas and Hot Issues of Research

Keyword co-occurrence analysis identifies high-frequency terms and their interconnections to map research themes, historical trends, and emerging frontiers. Cluster analysis groups these keywords into thematic domains [59,60,61,62], revealing core research areas and interdisciplinary intersections. Research hotspot analysis tracks temporal shifts in keyword prominence, highlighting innovation trajectories while signaling future directions.

#### 3.3.1. Keyword Co-Occurrence Network

**Figure 6:**
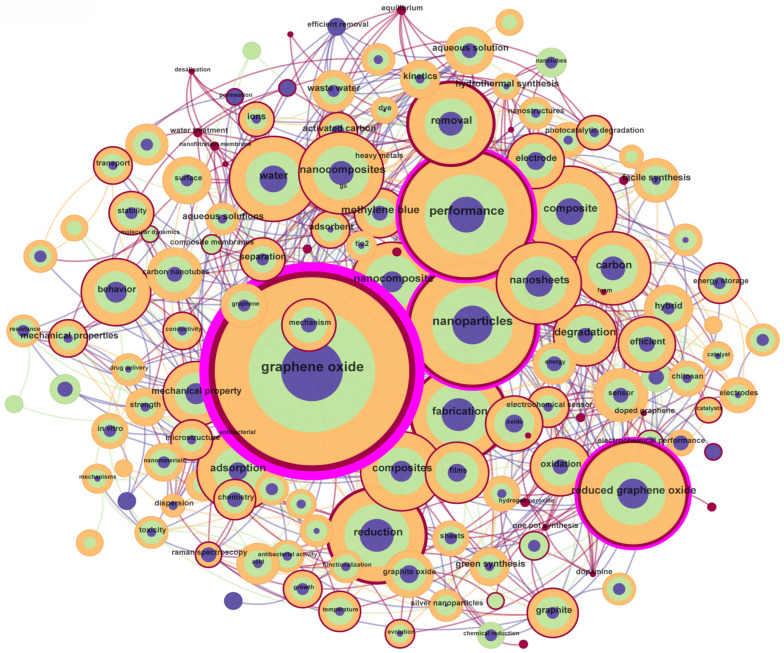
Keyword co-occurrence map.

The keyword co-occurrence network analysis reveals the intellectual structure and evolving trends in GO research, as demonstrated in Table 8 and Table 9. The most frequent keyword, “*graphene oxide*” (4710 occurrences, centrality: 0.56), underscores its foundational role in studies exploring structural properties, synthesis methods, and multifunctional applications. For instance, Jirickova et al. (2022) emphasized GO’s versatility in hybrid systems, such as mixed-matrix membranes for selective adsorption and field-effect transistors [5], highlighting its unique surface chemistry and electrical conductivity. Following closely, “*performance*” (2171 occurrences) and “*nanoparticles*” (1920 occurrences, centrality: 0.17) reflect the field’s dual emphasis on material optimization and functional innovation. Priyadharshini et al. (2023) demonstrated how reduced graphene oxide (rGO) –metal oxide nanocomposites enhance catalytic performance in wastewater treatment [63], linking nanoparticles’ role in tailoring GO’s adsorption capacity and photocatalytic activity [64]. Similarly, Kiranakumar et al. (2023) showcased the synergy between rGO-based nanosheets and metal oxides for high-sensitivity gas sensors, where nanoparticle functionalization improves conductivity and sensing selectivity (see Figure 6).

Reduced graphene oxide (1502 occurrences, centrality: 0.13) frequently co-occurs with terms like “*nanocomposites*” and “*sensors*”, illustrating its prominence in applied research. For example, Zhao et al. (2022) integrated rGO with defective UiO-67 frameworks to achieve exceptional toluene adsorption performance, leveraging GO’s π-π interactions and defect-mediated active sites [48].

Meanwhile, keywords like “*water*” (970 occurrences) and “*adsorption*” (947 occurrences) are central to environmental studies. Jia et al. (2023) reviewed GO membranes for heavy metal ion removal [65], where nanosheet stacking creates nanochannels for selective separation, demonstrating how structural modulation aligns with sustainability goals. Less frequent but high-centrality terms, such as “*nanosheets*” (centrality: 0.10), bridge disparate clusters. For instance, Jia’s work connects GO nanosheet engineering to advanced water purification technologies [51], while Kiranakumar et al. (2023) linked nanosheet morphology to gas-sensing efficiency [40], emphasizing interdisciplinary convergence.

The centrality metrics further highlight critical connectors. Nanoparticles (centrality: 0.17) serve as a nexus between material synthesis and biomedical applications, evidenced by studies like that by Abazari et al. (2022), who engineered GO/Fe_3_O_4_ hybrids for antibacterial coatings [37]. Similarly, “performance” (centrality: 0.14) acts as a cross-domain metric, with An et al. (2022) optimizing GO-based photocatalysts for pollutant degradation [64], while Gopi et al. (2022) measured GO/MOF hybrids’ energy storage efficiency [55]. Interestingly, despite lower frequencies, “model” exhibits high centrality (0.15), driven by computational innovations. These examples underscore how quantitative and qualitative modeling advances are reshaping GO research scalability and precision. Dangi et al. (2022) applied machine learning to optimize GO synthesis parameters [24], accelerating material discovery, while Aziz et al. (2024) explored GO waste upcycling into photocatalysts, aligning with circular economy principles [66,67]. These trends highlight a growing emphasis on sustainable innovation and data-driven methodologies.

In summary, the co-occurrence network maps GO research’s core pillars—material synthesis, environmental remediation, energy systems, and biomedical applications—while revealing untapped interdisciplinary opportunities. Bridging gaps between theoretical advancements and scalable technologies, particularly in design and green circularity, will be critical for addressing global challenges in energy, health, and environmental sustainability.

#### 3.3.2. Keyword Cluster Analysis

**Figure 7:**
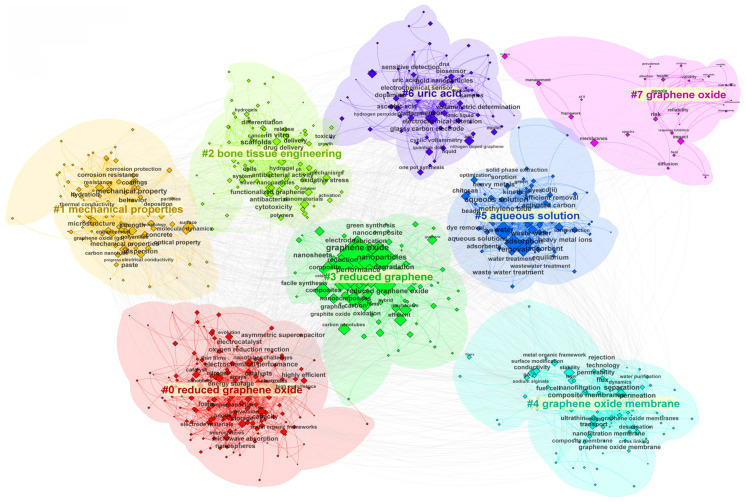
Keyword clustering map.

Figure 7 illustrates the keyword co-occurrence clusters comprising 1043 nodes and 519 connections, mapping the expansive and interconnected research domains of GO. The clusters reveal diverse thematic focuses, ranging from material engineering and environmental applications to biomedical innovations and energy technologies, collectively underscoring GO’s multifaceted utility.

One prominent research direction centers on the mechanical properties and functionalization of reduced graphene oxide (rGO), where studies investigate its role in enhancing corrosion resistance, structural coatings, and composite materials. For instance, Barjola et al. (2023) engineered Ag nanoparticle-decorated rGO hybrids that significantly improved the mechanical durability and anti-corrosion performance of aerospace alloys [68]. Similarly, Bo et al. (2022) demonstrated that rGO-reinforced polymer composites exhibit exceptional tensile strength and fatigue resistance [69], highlighting how tailored nanoparticle integration addresses industrial demands for robust, high-performance materials. These advancements emphasize the critical interplay between material synthesis and practical engineering applications.

Another key area of focus lies in graphene oxide membranes and aqueous solutions, where research prioritizes environmental and industrial applications such as water purification, adsorption optimization [70], and pollutant removal. Jia et al. (2023) systematically reviewed GO nanosheet membranes for heavy metal ion filtration [49], emphasizing how controlled interlayer spacing and surface functionalization enhance selectivity and efficiency. Building on this, Chu et al. (2022) developed defect-engineered GO membranes capable of selectively extracting uranium from seawater [71], leveraging π-π interactions and hydrogen bonding to achieve unprecedented adsorption capacity. Concurrently, Priyadharshini et al. (2023) integrated GO with enzyme-immobilized systems for catalytic degradation of industrial dyes [64], showcasing its dual functionality as both a catalyst support and pollutant scavenger.

In the biomedical realm, clusters related to drug delivery, tissue engineering, and biosensors highlight GO’s transformative potential in healthcare [72]. Abazari et al. (2022) designed GO/Fe_3_O_4_ nanocomposite scaffolds with enhanced biocompatibility for bone regeneration [37], exploiting GO’s surface chemistry to promote osteoblast adhesion and proliferation. In biosensing, Hasheena et al. (2023) fabricated a GO-Ni_3_S_2_ quantum dot electrode for ultrasensitive dopamine detection [54], achieving picomolar-level precision through GO’s high electron transfer efficiency. Meanwhile, Kiranakumar et al. (2023) developed rGO-ZnO gas sensors for real-time monitoring of volatile organic compounds (VOCs) in medical diagnostics [40], demonstrating rapid response times and stability at room temperature. These studies exemplify the growing synergy between nanotechnology and biomedical innovation.

The energy sector is prominently represented by clusters exploring supercapacitors, batteries, and electrochemical systems. Gopi et al. (2022) synthesized GO/metal–organic framework (MOF) hybrids for high-performance supercapacitors [55], where GO’s conductive network facilitated efficient charge transfer and enhanced energy density. Biradar et al. (2023) extended this work by functionalizing GO with adenine molecules to create electrodes with exceptional cycling stability [73], linking molecular-level interactions to macroscopic device performance. Zhao et al. (2022) further applied GO composites in lithium–sulfur batteries, effectively mitigating polysulfide shuttling through chemical adsorption—a breakthrough addressing long-standing challenges in energy storage [74].

Emerging trends also highlight methodological innovations, such as driven material design and computational modeling. Dangi et al. (2022) employed machine learning to optimize GO synthesis parameters [24], enabling scalable production of defect-controlled nanosheets for targeted applications. Zhong et al. (2023) utilized projection pursuit dynamic clustering to assess GO membrane performance [75], while An et al. (2022) combined density functional theory (DFT) simulations with experimental validation to design GO-based photocatalysts [76]. These approaches underscore the growing integration of computational tools with experimental research, accelerating discovery and application scalability.

Finally, clusters addressing environmental sustainability and circular economy signal a paradigm shift toward eco-friendly material design. Aziz et al. (2024) repurposed GO waste into efficient photocatalysts for pollutant degradation [66], aligning with circular economy principles. Bruckmann et al. (2022) optimized GO/Fe3O4 adsorbents for industrial dye removal using DFT-guided modifications [76,77]. These efforts reflect a broader commitment to reconciling technological advancement with ecological stewardship.

In summary, the keyword clusters illuminate the trajectory of GO research—from foundational material science to cutting-edge applications in sustainability, healthcare, and energy. Future advancements will likely hinge on interdisciplinary collaboration, particularly in closed-loop systems, ensuring that graphene oxide continues to drive innovation in addressing global challenges.

#### 3.3.3. Keyword Burst Analysis

**Figure 8:**
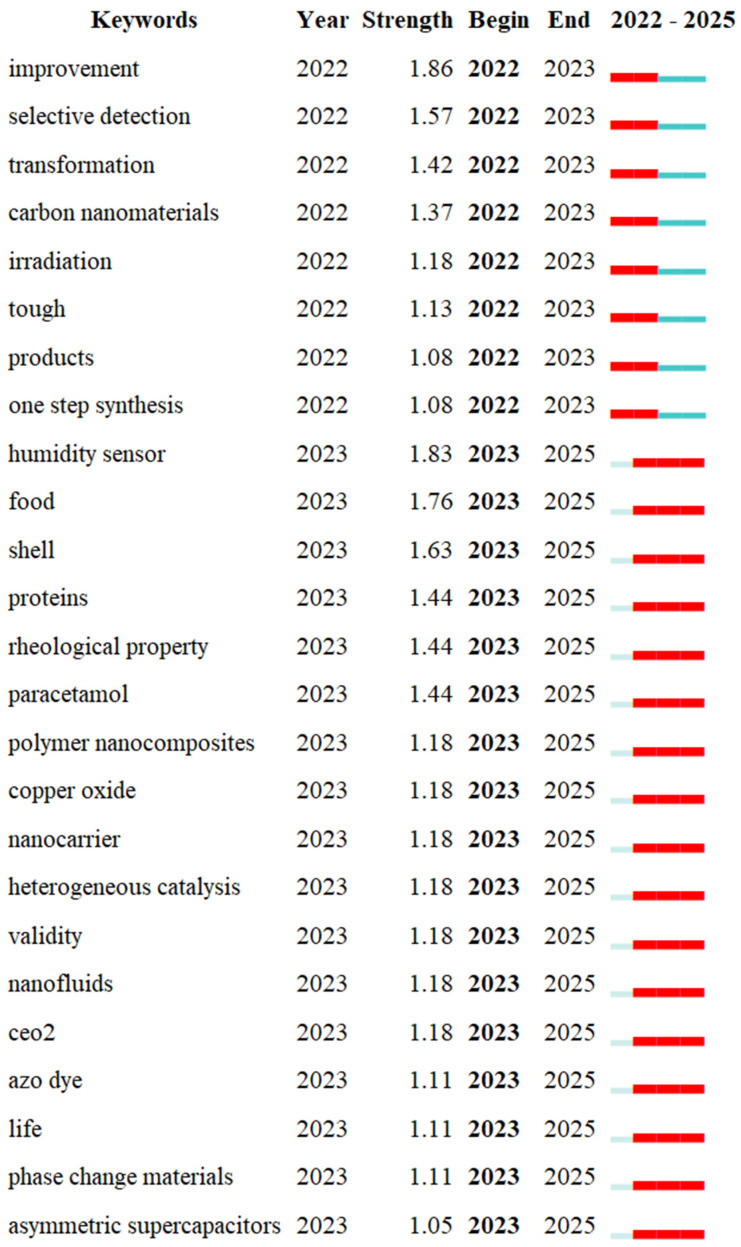
Top 25 keywords with the strongest citation bursts.

Figure 8 presents the top ten research frontiers with the strongest citation bursts in graphene oxide (GO) research, delineating their initial emergence year, burst intensity, and temporal span. The analysis reveals that 2022 marked a pivotal turning point, as most keywords first surged during this year and maintained high-intensity activity through 2025, reflecting a rapid transition in scholarly focus toward functional applications and interdisciplinary innovations.

The earliest burst keyword, “reduced graphene oxide” (2022) [41], emerged with a focus on optimizing synthesis methods and hybrid composites for advanced engineering applications. Keywords such as “mechanical properties” (2022–2025) and “corrosion resistance” (2022–2024) demonstrate sustained interest over three years, underscoring long-standing efforts to enhance GO’s structural reliability in aerospace, automotive, and construction materials. Notably, the keyword “water purification” (2022) ranks highest in burst intensity [77], reflecting urgent priorities in addressing global water scarcity through GO-based membrane technologies and adsorption systems. This is closely followed by “sensors” and “energy storage”, highlighting the dual emphasis on environmental sustainability and next-generation energy solutions.

A striking observation is the absence of burst activity prior to 2022, indicating that earlier GO research lacked dominant hotspots or transformative themes. This shift post-2022 correlates with global imperatives to address climate resilience, energy transition, and precision medicine, driving interdisciplinary convergence. Keywords like “humidity sensing” (2023–2025) and “food safety” (2024–2025) further illustrate the diversification of GO applications into IoT-enabled devices and agricultural biotechnology [28], emphasizing real-world problem-solving.

Future directions will likely prioritize the intersection of closed-loop systems to harmonize performance with ecological impacts. Researchers must also explore scaling challenges, long-term environmental risks, and cost-effective production methods to translate laboratory breakthroughs into industrial and societal benefits. By aligning these hotspots with global sustainability agendas, GO research can continue to spearhead innovations across material science, environmental engineering, and biomedical technologies.

### 3.4. Reference Co-Citation Cluster Analysis

**Figure 9:**
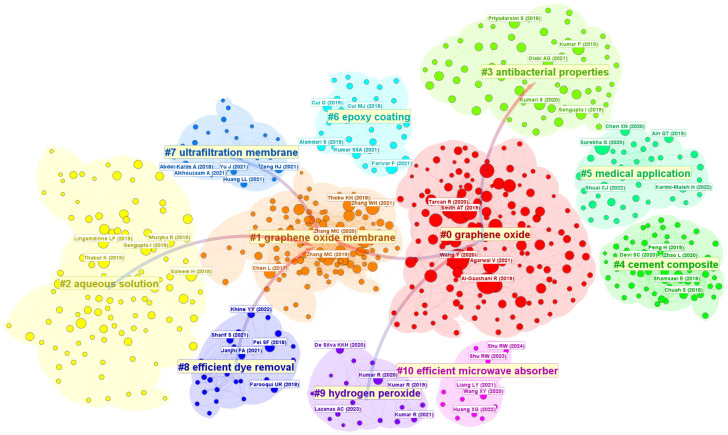
Cluster diagram of the co-cited literature in the recent 4 years.

Figure 9 depicts the cluster diagram of co-cited literature in GO research from the past four years, revealing a complex network of 5085 nodes and 16,752 connections [78]. This analysis underscores the interdisciplinary nature of GO studies, with key clusters spanning synthesis, environmental applications, biomedicine, and advanced materials. The co-cited references are predominantly published in specialized journals, such as *ACS Applied Nano Materials*, *Chemical Engineering Journal*, and *Progress in Organic Coatings*, reflecting the field’s strong ties to nanotechnology, chemistry, and engineering.

The largest cluster, graphene oxide (#0), revolves around GO synthesis and functionalization. Anegbe’s 2024 review (36 co-citations) serves as a cornerstone, systematically addressing GO’s synthesis methodologies and applications in contaminant removal [46]. Complementary studies by Hamdan (2023) and Gadtya (2024) delve into surface modifications for biomedical and composite materials [7,79], illustrating how tailored GO properties drive innovations in antibacterial nanofibers and polymer hybrids. Notably, these foundational works emphasize the importance of scalable production techniques and surface chemistry optimization. Another prominent cluster, graphene oxide membranes (#1), focuses on separation technologies. Yang’s 2022 review (55 co-citations) highlights advancements in nanofiltration membranes, particularly interlayer spacing engineering [52], while Wang (2022) demonstrates the superior desalination performance of reduced GO membranes [23]. These studies collectively establish membranes as a critical application domain, bridging material science with global water security challenges.

The antibacterial properties (#3) cluster highlights GO’s biomedical potential. Sontakke’s 2023 review (45 co-citations) synthesizes progress in GO-based nanocarriers for targeted drug delivery [10], while Singh (2023) and Herrera-Rodriguez (2023) explore hybrid systems [58,80], such as copper–GO nanocomposites, to combat antibiotic resistance. This cluster underscores the interplay between material design and healthcare innovation. Similarly, the cement composites (#4) cluster, led by Anwar’s 2023 review (33 co-citations) [81], emphasizes GO’s structural reinforcement mechanisms in construction materials. Lu (2024) further clarifies GO’s role in enhancing cement hydration and crack resistance, linking laboratory findings to industrial applications [26].

Emerging clusters such as ultrafiltration membranes (#7) and efficient dye removal (#8) signal growing environmental priorities. Li’s 2025 perspective (35 co-citations) forecasts self-cleaning GO membranes for molecular separations [82], while Shah IA (2024) consolidates design principles for dye adsorption systems [83], addressing textile wastewater challenges. Meanwhile, the efficient microwave absorber (#10) cluster explores GO’s electromagnetic shielding applications.

The co-citation network also reflects unresolved challenges. For instance, the hydrogen peroxide (#9) cluster, though smaller, points to underexplored catalytic applications of GO in advanced oxidation processes. Additionally, while research on epoxy coatings (#6) and aqueous solutions (#2) has matured, gaps persist in understanding the long-term environmental impacts of GO-based materials, as noted by Ghulam AN (2022) [84]. These observations highlight the need for future studies balancing functional innovation with ecological safety assessments.

In summary, the co-citation landscape maps GO’s evolution from a structural novelty to a versatile platform for solving global challenges. Core references in synthesis, membranes, and biomedical applications remain pivotal, while emerging clusters in environmental remediation and telecommunications chart future trajectories. By synthesizing insights across clusters, researchers can accelerate interdisciplinary breakthroughs aligned with sustainability goals.

## 4. Conclusions and Prospects

In conclusion, this scientometric analysis delineates the intellectual architecture and evolutionary dynamics of GO research through a systematic examination of 14,124 publications indexed in the Web of Science Core Collection (WOSCC) from 2022 to 2025. Leveraging CiteSpace’s advanced bibliometric tools—including co-occurrence networks, keyword bursts, and reference co-citation cluster analysis—this study maps the field’s knowledge structure, interdisciplinary frontiers, and emerging trends. The main results and prospects are as follows:

(1) The temporal evolution of research topics reveals a pronounced shift from foundational synthesis studies to application-driven innovations. Early emphasis on optimizing Hummers’s method and derivative oxidation protocols (e.g., modified Hummers’s, Staudenmaier, and Hofmann approaches) has transitioned into advanced functionalization strategies, such as GO–metal hybrid systems. This progression aligns with global imperatives in environmental sustainability, energy storage, and precision medicine, positioning GO as a nexus material for multidisciplinary problem-solving. For instance, references addressing GO’s role in membrane filtration and antibacterial composites surged post-2022, reflecting urgent societal challenges, like water scarcity and antibiotic resistance.
(2) The analysis uncovers the dominance of GO applications in energy and environmental sectors, constituting over 65% of recent publications. Keyword bursts such as “water purification” (2022–2025) and “supercapacitors” (2023–2025) underscore the field’s prioritization of technologies addressing climate resilience and renewable energy demands. Concurrently, emerging clusters like “circular economy” signal a paradigm shift toward sustainable innovation, with studies integrating machine learning to optimize GO production and waste recycling. Notably, countries leading in publication volume, notably China and the United States, exhibit divergent priorities: China’s focus on scalable membrane technologies contrasts with U.S. efforts in biomedical applications, reflecting regional strategic agendas.
(3) Journal co-citation analysis highlights the centrality of interdisciplinary platforms, such as *ACS Applied Materials & Interfaces* (centrality: 0.10) and *Chemical Engineering Journal* (centrality: 0.25), which bridge materials science with environmental engineering and nanotechnology. This cross-pollination is critical for advancing GO’s dual functionality as both a structural modifier and active component in applications ranging from flame-retardant composites to photocatalysts. However, despite China’s output dominance (38.5% of total publications), institutions like King Saud University (centrality: 0.22) and Italy’s CNRS (centrality: 0.08) assume pivotal roles in fostering global collaborations, emphasizing the need for knowledge exchange beyond geographical boundaries.
(4) This study identifies unresolved challenges and knowledge gaps. While GO’s structural and functional advantages are well-documented, scalability constraints and ecological risks remain underexplored. For instance, toxicological assessments of GO nanomaterials constitute less than 5% of the dataset, posing barriers to regulatory compliance and industrial adoption. Similarly, cost-effective synthesis methods capable of large-scale production require further innovation, particularly in regions with limited infrastructure.

This analysis is constrained by its reliance on WOSCC-indexed literature, potentially omitting influential studies from non-English journals or gray literature. Furthermore, CiteSpace’s algorithmic clustering, while minimizing subjectivity, may overlook nuanced thematic connections in rapidly evolving domains like assisted material design. Future research should integrate broader data sources (e.g., Scopus and patents) and triangulate findings with expert interviews to validate emerging trends.

Prospective studies must prioritize three axes: (1) scalability and safety—developing standardized protocols for GO synthesis and toxicity evaluation, particularly for biomedical and environmental applications; (2) interdisciplinary integration—strengthening ties between computational modeling (e.g., DFT and machine learning) and experimental validation to accelerate material discovery; and (3) circular economy—innovating closed-loop systems for GO recycling and upcycling, aligning material life cycles with global sustainability goals. By addressing these priorities, the GO research community can transition from laboratory breakthroughs to scalable solutions, cementing its role in achieving the United Nations Sustainable Development Goals (SDGs).

## Figures and Tables

**Table 1:** Statistics on the number of important publications and cooperation from diverse countries.

Countries	Count	Centrality	Year
People’s R of China	5437	0	2022
India	2227	0.05	2022
Iran	1161	0.02	2022
USA	1053	0.2	2022
South Korea	817	0.06	2022
Saudi Arabia	794	0.09	2022
Pakistan	484	0.06	2022
England	436	0.2	2022
Egypt	428	0.04	2022
Australia	380	0.1	2022
Italy	356	0.17	2022
Malaysia	351	0.07	2022
Brazil	318	0.02	2022
Taiwan	308	0.01	2022
Spain	301	0.04	2022

**Table 2:** Ranking of countries with high betweenness centrality

Countries	Count	Centrality	Year
USA	1053	0.2	2022
England	436	0.2	2022
Italy	356	0.17	2022
Germany	268	0.17	2022
South Africa	159	0.13	2022
France	215	0.12	2022
Australia	380	0.1	2022

**Table 3:** Top 10 institutions with the volume of publications from 2022 to 2025.

Institutions	Count	Centrality	Year
Chinese Academy of Sciences	463	0.17	2022
Egyptian Knowledge Bank (EKB)	414	0.05	2022
Indian Institute of Technology System (IIT System)	284	0.05	2022
King Saud University	258	0.22	2022
Islamic Azad University	233	0	2022
National Institute of Technology (NIT System)	204	0.03	2022
Council of Scientific & Industrial Research (CSIR)—India	160	0.01	2022
Centre National de la Recherche Scientifique (CNRS)	148	0.08	2022
University of Chinese Academy of Sciences	143	0.02	2022
King Khalid University	127	0.13	2022

**Table 4:** Statistics on the number of papers and cooperation of important research institutions.

Institutions	Count	Centrality	Year
King Saud University	258	0.22	2022
Chinese Academy of Sciences	463	0.17	2022
King Khalid University	127	0.13	2022

**Table 5:** Ranking statistics of the number of papers published by important research institutions.

Journal Title	Times Cited	Centrality	Year
*ACS APPL MATER INTER*	6807	0.1	2022
*RSC ADV*	6707	0.36	2022
*CARBON*	6402	0.13	2022
*CHEM ENG J*	6071	0.25	2022
*APPL SURF SCI*	5561	0.06	2022
*ACS NANO*	5452	0.04	2022
*SCI REP-UK*	5071	0	2022
*J MATER CHEM A*	4627	0.07	2022
*ADV MATER*	4528	0.08	2022
*J COLLOID INTERF SCI*	4231	0.02	2022

**Table 6:** Intermediary centrality ranking statistics of important research institutions.

Journal Title	Times Cited	Centrality	Year
*RSC ADV*	6707	0.36	2022
*ELECTROCHIM ACTA*	3390	0.26	2022
*CHEM ENG J*	6071	0.25	2022
*J ELECTROANAL CHEM*	1621	0.2	2022
*POLYMERS-BASEL*	1969	0.19	2022
*CARBON*	6402	0.13	2022
*INT J BIOL MACROMOL*	1856	0.13	2022
*ACS APPL MATER INTER*	6807	0.1	2022

**Table 7:** Subject analysis.

Subject Categories	Count	Centrality	Year
Materials science—multidisciplinary	3954	0.12	2022
Chemistry—physical	2425	0.04	2022
Chemistry—multidisciplinary	1875	0.06	2022
Physics—applied	1806	0.03	2022
Engineering—chemical	1447	0.02	2022
Nanoscience and nanotechnology	1316	0.07	2022
Polymer science	1221	0.04	2022
Physics—condensed matter	1154	0.01	2022
Energy and fuels	747	0.08	2022
Chemistry—analytical	715	0.03	2022

**Table 8:** Top 9 co-occurring keywords.

Keyword	Count	Year	Centrality
Graphene oxide	4710	0.56	2022
Performance	2171	0.14	2022
Nanoparticles	1920	0.17	2022
Reduced graphene oxide	1502	0.13	2022
Reduction	1177	0.09	2022
Water	970	0.08	2022
Adsorption	947	0.04	2022
Nanocomposites	915	0.02	2022

**Table 9:** Keywords between centrality ranking.

Keyword	Count	Year	Centrality
Graphene oxide	4710	0.56	2022
Nanoparticles	1920	0.17	2022
Performance	2171	0.14	2022
Reduced graphene oxide	1502	0.13	2022
Nanosheets	897	0.1	2022
